# Accelign: a GPU-based library for accelerating pairwise sequence alignment

**DOI:** 10.1186/s12859-026-06521-0

**Published:** 2026-06-21

**Authors:** Felix Kallenborn, Fawaz Dabbaghie, Martin Steinegger, Bertil Schmidt

**Affiliations:** 1 Department of Computer Science, Johannes Gutenberg University, Mainz, 55099 Germany; 2 School of Biological Sciences, Seoul National University, Seoul, 08826 Republic of Korea

**Keywords:** Pairwise alignment, GPU, DNA, RNA, Protein sequences, Dynamic programming, Parallelization

## Abstract

**Background:**

The continually increasing volume of sequence data results in a growing demand for fast implementations of core algorithms. Computation of pairwise alignments based on dynamic programming is an important part in many bioinformatics pipelines and a major contributor to overall runtime due to the associated quadratic time complexity. This motivates the need for a library of efficient implementations on modern GPUs for a variety of alignment algorithms for different types of sequence data including DNA, RNA, and proteins.

**Results:**

Accelign is a library of accelerated pairwise sequence alignment algorithms for CUDA-enabled GPUs. Its parallelization strategy is based on a common wavefront design that can be adapted to support a variety of dynamic programming algorithms: local, global, and semi-global alignment of genomic and protein sequences with a variety of commonly used scoring schemes supporting one-to-one, one-to-many or all-to-all pairwise sequence alignments. This leads to a peak performance between 16.1 TCUPS and 9.1 TCUPS for computing optimal global alignment scores with linear gaps and affine gap penalties on a single RTX PRO 6000 Blackwell GPU, respectively. In addition, our library demonstrates significant speedups in several real-world case studies over prior CPU-based (SeqAn, Parasail, BSalign, EdLib, KSW2, WFA2, A*PA2) and GPU-based libraries (ADEPT, GASAL2), and can even outperform highly customized algorithms (WFA-GPU, CUDASW++4.0). Furthermore, the performance of our approach scales linearly with the number of employed GPUs, which makes it feasible to exploit multi-GPU nodes for increased processing speeds.

**Conclusion:**

Accelign provides significant speedups for commonly used pairwise alignment algorithms compared to prior implementations. It is freely available at https://github.com/fkallen/Accelign.

## Background

Over the past decade, the life sciences have seen an unprecedented surge in data volume, largely fueled by rapid advancements in high-throughput sequencing technologies. Corresponding datasets typically contain large amounts of short reads (of a few hundred base-pairs in length for Illumina platforms), long reads (varying between a few thousands to hundreds of thousand base-pairs in length for Nanopore-based sequencers), or protein sequence collections (varying between a few and tens of thousands of amino acids). Corresponding pipelines frequently use dynamic programming (DP) algorithms to compute batches of pairwise alignments with typical examples including local, global, or semi-global sequence alignments for DNA/RNA read mapping or protein homology search. Associated running time grows with the product of the two sequence lengths. As a consequence, pairwise alignment computations often dominate the overall execution time of modern bioinformatics tools. Therefore, their highly efficient implementation is necessary. This motivates the need for solutions that can accelerate a variety of these algorithms on modern parallel computing platforms which can keep pace with the increasing volume of sequence data. To handle the computational intensity of sequence alignment, researchers have developed parallel implementations optimized for various hardware platforms - including CPUs [1–12], GPUs [13–25], FPGAs [12, 26–29], Processing-in-Memory (PIM) architectures [30], and even custom ASICs [31–33]. These tools generally serve two main use cases:

- **Aligning very long sequences:** Focused on computing a single pairwise alignment between large sequences such as entire chromosomes or genome
- **Processing large batches of short alignments:** Designed to compute many independent pairwise alignments for aligning short DNA/RNA reads or protein sequences.

Dominant parallelization strategies include inter-sequence parallelism (runs multiple independent alignment jobs simultaneously), intra-sequence parallelism (breaks down the DP matrix of a single alignment into parallelizable units), and hybrid parallelism (combines both approaches to fully exploit modern hardware capabilities).There are several CPU-based libraries that provide multi-threaded and SIMD vectorized implementations of a variety of alignment algorithms including Seqan [1], SSW [5], Parasail [4], BSalign [7], EdLib [8], and KSW2 [10]. In comparison, existing GPU-based alignment libraries are often limited in their functionality; e.g. ADEPT [19] only support local alignment of both DNA and protein sequences while GASAL2 [18] is limited to DNA sequences. Although, NVBIO [20] provides more functionality it has not been updated since 2015 and thus does not support modern GPU architectures. In addition, there are a number of more recent specialized GPU-based implementations. For example, WFA-GPU [34] is an algorithm for global alignment of DNA sequences under a certain type of scoring scheme. $\hbox {G}^3$SA [35] delivers a complete GPU-accelerated pipeline for aligning both short and long DNA reads. Its pairwise alignment module adopts a warp-per-alignment strategy, assigning a single thread to compute one column of the DP matrix during seed extension. Because each thread performs only a few arithmetic operations for every byte fetched from global memory, the implementation exhibits a low compute-to-memory ratio. Moreover, the frequent transfers to and from auxiliary buffers further increase memory traffic, which limits overall efficiency for typical read lengths.

We have recently presented CUDASW++4.0 [21] and MMSeqs2-GPU [22] for protein sequence database search based on computing local pairwise alignments. While CUDASW++4.0 is based on the Smith-Waterman for computing optimal alignments with affine gap penalties, MMSeqs2-GPU additionally uses a gapless pre-filter heuristic. We have shown that our novel parallelization strategy based on warp intrinsics for fast inter-thread communication and minimization of overall executed instructions can outperform prior approaches and achieve close-to-peak performance on modern GPU architectures. While this method is highly efficient, it has so far been limited to the use-case of protein homology search.

Current GPU-accelerated alignment tools exhibit significant limitations in scope, being narrowly optimized for specific sequence types (e.g., nucleotide vs. protein), alignment paradigms (local, global, semi-global), scoring schemes, and use cases. For instance, protein database homology searches require profile-based scoring with position-specific substitution matrices per query residue, whereas NGS read alignment typically employs simplified nucleotide match/mismatch scoring. These divergent computational demands necessitate fundamentally different memory access patterns-a critical factor for GPU efficiency. Furthermore, existing implementations lack flexibility: modifying them to support alternative data types (e.g., transitioning from protein homology searches to DNA read mapping) or alignment classes (e.g., semi-global alignments for *denovo* assembly overlap detection) often requires non-trivial architectural changes.

To address these constraints, we generalize our parallelization framework to accelerate diverse pairwise alignment algorithms across genomics and proteomics workflows. We demonstrate how a unified core algorithm can be specialized for heterogeneous scenarios through configurable scoring mechanisms. Introducing Accelign (Accelerate and align), our novel GPU library implements optimized strategies for substitution table and position-specific scoring matrix (PSSM) lookups, strategically balancing shared memory utilization, instruction throughput, and access latency according to application-specific requirements. This leads to a new library containing the following features:

- **Sequence data types:** Accelign supports various alphabets such as those for DNA, RNA, and protein.
- **Alignment classes:** Accelerated kernels for local, global, and semi-global alignment are supported. Note that our kernels perform score-only computation but no traceback. However, for local alignment and semi-global alignment the start and the end-position can be output in addition to the score.
- **Scoring schemes:** We provide optimized kernels for linear and affine gap penalties. In addition arbitrary nucleotide or amino acid substitution matrices can be used or alternatively position-specific scoring matrices (PSSMs).
- **Application scenarios:** We support one-to-one alignments between two given sets of sequences, one-to-many alignments between a query and a set of sequences, and all-to-all alignments between two sets of sequences.
- **Multi-GPU:** Higher speeds can be achieved by using multiple GPUs connected to the same host by distributing the utilized datasets.
- **APIs:** The library is written CUDA C++ and we provide interfaces for its usage with C++ .

We initially evaluate the theoretical peak performance of our various kernels using simulated datasets on different GPU architectures in terms of CUPS (Cell Updates Per Second). Subsequently, we evaluate the runtime performance of Accelign using real-world datasets consisting of Illumina reads and protein sequence databases. Our performance comparison shows that Accelign consistently outperforms other GPU-based libraries (GASAL2, ADEPT) and CPU-based libraries (SeqAn, Parasail, BSalign, EdLib, KSW2). In addition, it is faster than state-of-the-art specialized GPU-based implementations such as WFA-GPU for global alignment of Illumina reads and CUDASW++4.0 for protein database scanning. Even higher speeds can be achieved by using multiple GPUs connected to the same host with close-to-linear scaling. Last, we show the potential of an exact GPU-accelerated aligner in the context of read mapping for evolutionary divergent sequences.

Table 1 compares the features of all GPU-based pairwise alignment implementations considered in our performance evaluation. To summarize, Accelign not only performs best in our tests but also provides the highest flexibility.

**Table 1:** Feature comparison of all tested GPU-based pairwise alignment implementations

		Accelign	GASAL2	ADEPT	WFA-GPU	CUDASW4.0
Alphabet	DNA/RNA	✓	✓	✓	✓	✕
	Protein	✓	✕	✓	✕	✓
Scoring scheme	Linear gap ^1^	✓	✕	✕	✕	✕
	Affine gap	✓	✓	✓	✓	✓
	PSSM	✓	✕	✕	✕	✕
Alignment type	Global	✓	✓	✓	✓	✕
	Local	✓	✓	✓	✕	✓
	Semi-global	✓	✓	✕	✕	✕
	Endpoints ^2^	✓	✓	✕	✕	✕
	Trace-back	✕	✓	✓	✓	✕
Use-cases	Input on device ^3^	✓	✕	✕	✕	✓
	1-1	✓	✓	✓	✓	✕
	1-all	✓	✓	✕	✕	✓
	all-all	✓	✓	✕	✕	✓
	Multi-GPU^4^	✓	✓	✕	✕	✓

Indicates dedicated API for linear gap penalties. Can be emulated with affine gap penalties at the cost of performance

Indicates dedicated API for endpoints without trace-back computation

API accepts sequences which already reside in GPU memory

Indicates possibility of simultaneously using multiple GPUs in parallel. Not possible if, for example, only a synchronous API is provided

### Pairwise alignment computation

Pairwise sequence alignment compares two sequences $S = (s_1 s_2 \ldots s_n)$ of length *n* and $Q = (q_1 q_2 \ldots q_m)$ of length *m* over an alphabet $\Sigma$. For each character pair $(s_i, q_j)$, the algorithm determines whether to align the characters or insert a gap, recording the optimal score in a DP matrix *H*. The recurrence relation is defined as:

1 $$\begin{aligned} H(i,j) = \max {\left\{ \begin{array}{ll} H(i-1,j-1) + \sigma (s_i,q_j) \\ E(i,j) \\ F(i,j) \\ \nu \\ \end{array}\right. } \quad \begin{array}{c} 1 \le i \le n \\ 1 \le j \le m \end{array} \end{aligned}$$

where $\sigma : \Sigma \times \Sigma \rightarrow \mathbb {Z}$ is a substitution score function, and $\nu$ distinguishes alignment types (explained below). The entry *H*(*i*, *j*) represents the optimal alignment score for prefixes $(s_1 \ldots s_i)$ and $(q_1 \ldots q_j)$ under the chosen scoring scheme.

For a *linear gap penalty* with cost $\alpha$:

2 $$\begin{aligned} E(i,j)&= H(i-1,j) - \alpha \end{aligned}$$

3 $$\begin{aligned} F(i,j)&= H(i,j-1) - \alpha \end{aligned}$$

*Affine gap penalties* use distinct costs for gap opening ($\alpha$) and extension ($\beta$), penalizing a gap of length *k* as $\alpha + (k-1)\beta$:

4 $$\begin{aligned} E(i,j)&= \max {\left\{ \begin{array}{ll} E(i-1,j) - \beta \\ H(i-1,j) - \alpha \end{array}\right. } \end{aligned}$$

5 $$\begin{aligned} F(i,j)&= \max {\left\{ \begin{array}{ll} F(i,j-1) - \beta \\ H(i,j-1) - \alpha . \end{array}\right. } \end{aligned}$$

Initialization of *H*, *E*, and *F* as well as the location of the optimal score depends on the alignment type:

- **Local alignment** ($\nu = 0$): Finds the highest-scoring pair of substrings. Matrices initialize as $H(0,0) = H(i,0) = H(0,j) = 0$, $E(0,0) = F(0,0) = F(i,0) = E(0,j) = -\infty$, $E(i,0) = -\alpha -(i-1) \cdot \beta$, and $F(0,j) = -\alpha - (j-1) \cdot \beta$.
- **Global alignment** ($\nu = -\infty$): Aligns entire sequences. Optimal score is *H*(*n*, *m*) with initialization $H(0,0) = 0$, $H(i,0) = E(i,0) = -\alpha - \beta (i-1)$, $H(0,j) = F(0,j) = -\alpha - \beta (j-1)$, $E(0,0) = E(0,j) = F(0,0) = F(i,0) = -\infty$.
- **Semi-global alignment** ($\nu = -\infty$): Does not penalize gaps at the beginning and the end of the alignment. This leads to the same specifications as for local alignment with two exceptions: (i) $\nu = -\infty$, and (ii) the optimal score is determined as the maximal value in the last row or column of *H*.

**Fig. 1:**
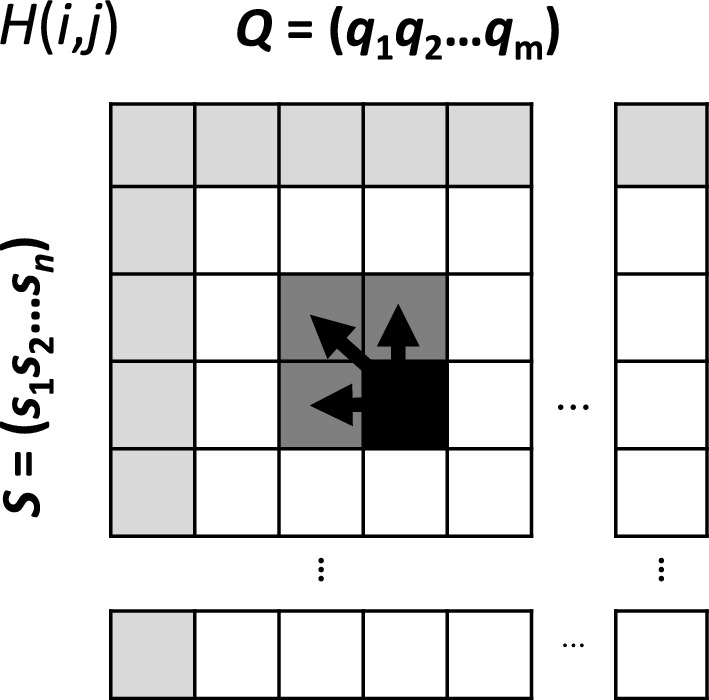
Conceptual layout and dependency relationships of the DP matrix *H*. Light gray cells denote initialization values, while dark gray cells indicate the three immediate predecessor subproblems (top, left, and diagonal neighbors) required to compute the active cell (black). Arrows illustrate the direction of dependency propagation during matrix filling

Figure 1 illustrates the conceptual layout and dependency relationship between cells of *H*. Note that score-only computations of all these variants can be performed in linear space ${\mathcal {O}}(\min \{n, m\})$ and quadratic time ${\mathcal {O}}(n \cdot m)$.

### Position specific scoring matrices

A PSSM (Position Specific Scoring Matrix) – also known as a PWM (position weight matrix) or a profile – is a frequently used representation in which substitution scores are given separately for each position in a protein or DNA profile; i.e., it features one row per symbol of the considered alphabet $\Sigma$ and one column per position of the profile. PSSMs are often obtained from multiple sequences alignments (MSAs) and are for example often used for homology search in protein sequence databases or for DNA motif discovery.

Typical tasks require computing pairwise alignments between a sequence $S=(s_1 s_2 \ldots s_n)$ of length *n* and a PSSM *Q* represented by a matrix of size $|\Sigma |\times m$; i.e., the value $Q[s_i,j]$ represents the likelihood of observing $s_i \in \Sigma$ at position $1 \le j \le m$. The optimal alignment score can be computed by replacing the lookup $\sigma (s_i,q_j)$ in Eq. 1 by the lookup $Q[s_i,j]$.

Note that storing the PSSM generally requires more data than the substitution function. Thus, fast memory access to the PSSM is key to achieve high efficiency for computing pairwise alignments between sequences and PSSMs.

#### Use case scenarios

Bioinformatics applications that require large amounts of pairwise alignments typically consider two given sets ${\mathcal {R}} = \{ R_1,..., R_N\}$ and ${\mathcal {T}} = \{ T_1,..., T_M\}$ containing *N* and *M* sequences, respectively.

#### One-to-one

For this use case holds $N=M$ and refers to computing an alignment between $R_i$ and $T_i$ for all $1 \le i \le N$ resulting in a total of *N* pairwise alignment computations. This is a common task in various applications including read mapping. We will consider such alignments of Illumina reads in our first case study.

#### One-to-all

This corresponds to the special case where one of the sets contains only a single sequence; i.e., $N=1$. Thus, $R_1$ is aligned to all sequences in ${\mathcal {T}}$ resulting in a total of *M* pairwise alignments. This task occurs in database search applications such as protein homology searches (which we consider in our second case study) or when aligning a set of reads against an evolutionary divergent reference such as HIV (which we consider in our second case study).

#### All-to-all

This use case refers to the task of aligning each sequence in ${\mathcal {R}}$ to each sequence in ${\mathcal {T}}$ resulting in a total of $N \times M$ alignments. It can be expressed as multiple one-to-all alignments. This task occurs for example in homology search with a set of queries, in distance matrix computation for progressive multiple sequence alignment, or when aligning a set of reads against a set of references such as tRNAs.

#### Architectural features for dynamic programming on GPUs

CUDA kernels execute as grids of thread blocks, each mapped to a single streaming multiprocessor (SM). Within each block, threads are grouped into warps (32 threads), which execute in lockstep (SIMT execution model). Modern GPUs provide a memory hierarchy where global memory offers high capacity but high latency, while on-chip shared memory and constant memory provide lower latency access. The fastest data access occurs through thread-local registers, and modern architectures enable intra-warp communication via warp-level primitives by performing direct register-to-register data exchange without memory transactions.

Our implementation leverages __shfl_down_sync() and __shfl_up_sync() warp shuffle instructions to minimize memory traffic. For example:

R1 = __shfl_up_sync(0xFFFFFFFF, R0, 1, 32);

transfers the value in register R0 from thread *i* to register R1 in thread $i+1$ within each warp ($0 \le i < 31$).

Recent GPU architectures introduce specialized instructions critical for accelerating DP:

- **Half2 Arithmetic (Ampere+)**: Enables dual half-precision (FP16) operations per 32-bit instruction. Crucially, Ampere and later architectures support half2 min/max comparisons required for alignment recurrence relations, effectively doubling throughput for score computations.
- **Max3 Instructions (Hopper, Blackwell)**: Introduces vhmnmx instructions for three-input min/max operations on half2 data, accelerating recurrence evaluations requiring comparisons across multiple predecessor cells.
- **Dynamic Programming eXtension (DPX) (Hopper, Blackwell)**: Provides integer-based instructions (vimax3, viaddmax) that fuse comparison and arithmetic operations for 16/32-bit integers. These support all combinations of min/max with add/sub operations (including ReLU clamping), directly accelerating the core recurrence relations of sequence alignment algorithms.
- **8-bit integer support (Blackwell)**: CUDA 13.2 unlocked native hardware instructions on Blackwell (compute capability 12) for (saturated) add/sub and min/max for four 8-bit integers packed into a 32-bit word.

The following section details algorithmic adaptations leveraging these features to achieve peak performance in our GPU-accelerated alignment library.

## Implementation

### Core parallel algorithm

Our parallelization strategy assigns each pairwise alignment to a **cooperative group (CG)** – a synchronized thread subgroup (of size $p \in \{4, 8, 16, 32\}$) executing in lockstep with warp-level communication capabilities. Within a thread block, multiple CGs process independent alignments concurrently, while threads in a single CG cooperatively compute cells for one alignment’s DP matrix.

As established in Eqs. 1–5 and Fig. 1, each DP cell depends on its left, top, and top-left neighbors, enforcing a strict topological order for computation. This dependency structure – consistent across all alignment variants in our library – enables parallel evaluation along minor diagonals (termed **wavefronts**).

Accelign’s core algorithm computes a single alignment within a CG as follows: Given sequences $S = (s_1 \ldots s_n)$ (length *n*) and $Q = (q_1 \ldots q_m)$ (length *m*), we partition the DP matrix into *p* segments of *k* columns each ($m = p \cdot k$ without loss of generality).

**Fig. 2:**
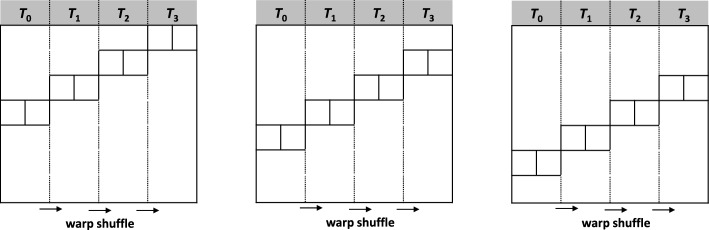
Three iterations of wavefront-based DP computation using $p=4$ threads, each assigned $k=2$ columns. Each thread computes *k* new cells in the current wavefront *d* using values from the immediately preceding wavefront ($d-1$), and from the wavefront two steps prior ($d-2$), all maintained in thread-local registers. Boundary values (rightmost cell in each thread’s segment) are exchanged via warp shuffle operations as indicated by arrows

Due to hardware constraints, performance will drop significantly for larger *k*, say $k > 32$ or $k > 64$, depending on the algorithm. Thus, for long queries, the matrix will be partitioned into *x* tiles of size $p \cdot k$ where $m \le x \cdot p \cdot k$. This tiling approach is further explained in a latter section. Each thread $T_t$ ($0 \le t < p$) computes *k* contiguous cells per wavefront. Over $n + p - 1$ iterations, thread $T_t$ processes cells along minor diagonal *i* at row $i - t$. Crucially, all values from the current and prior wavefronts reside in thread-local registers. Boundary communication –where $T_t$ accesses the rightmost cell computed by $T_{t-1}$ in the previous wavefront – is implemented via low-latency warp shuffles (__shfl_up_sync()). This mapping strategy is illustrated in Fig. 2.

Listing 1 shows pseudocode of a GPU device function for the described core algorithm which is called by each CG to compute a different global alignment with a linear gap penalty using float 32-bit arithmetic. Template parameters are used to define the number of DP matrix columns assigned to each thread (k) and the CG size (p). Thus, by changing the definitions of *p* and *k*, kernels tailored for different sequence sizes can be generated. Prior to the wavefront loop, we transfer *Q* to thread-local registers (QReg[]) and initialize the DP matrix values stored in each thread in the registers M[], M_left, M_diag in initDP(). processDP() computes *k* DP cells per thread in each wavefront iteration (highlighted cells in Fig. 2) using only thread-local in-register computation and shared memory lookups to the PSSM or substitution function (as will be explained in the corresponding subsection below). The rightmost current DP cell of each thread stored M[k] is then communicated to Register M_left of the neighboring thread using a warp shuffle-up instruction. Every *p* iteration steps, we load one value from *S* per thread to Register SReg_cache, while Register SReg always stores the character from *S* needed in each iteration step. A warp shuffle-up and a warp shuffle-down update SReg_cache and SReg, respectively, with characters from neighboring threads.

In the following we will describe how this basic kernel can be extended to implement the various pairwise alignment variants considered in Accelign.

**Listing 1** Pseudo-code of our core parallel wavefront algorithm for linear global

**Figure:**
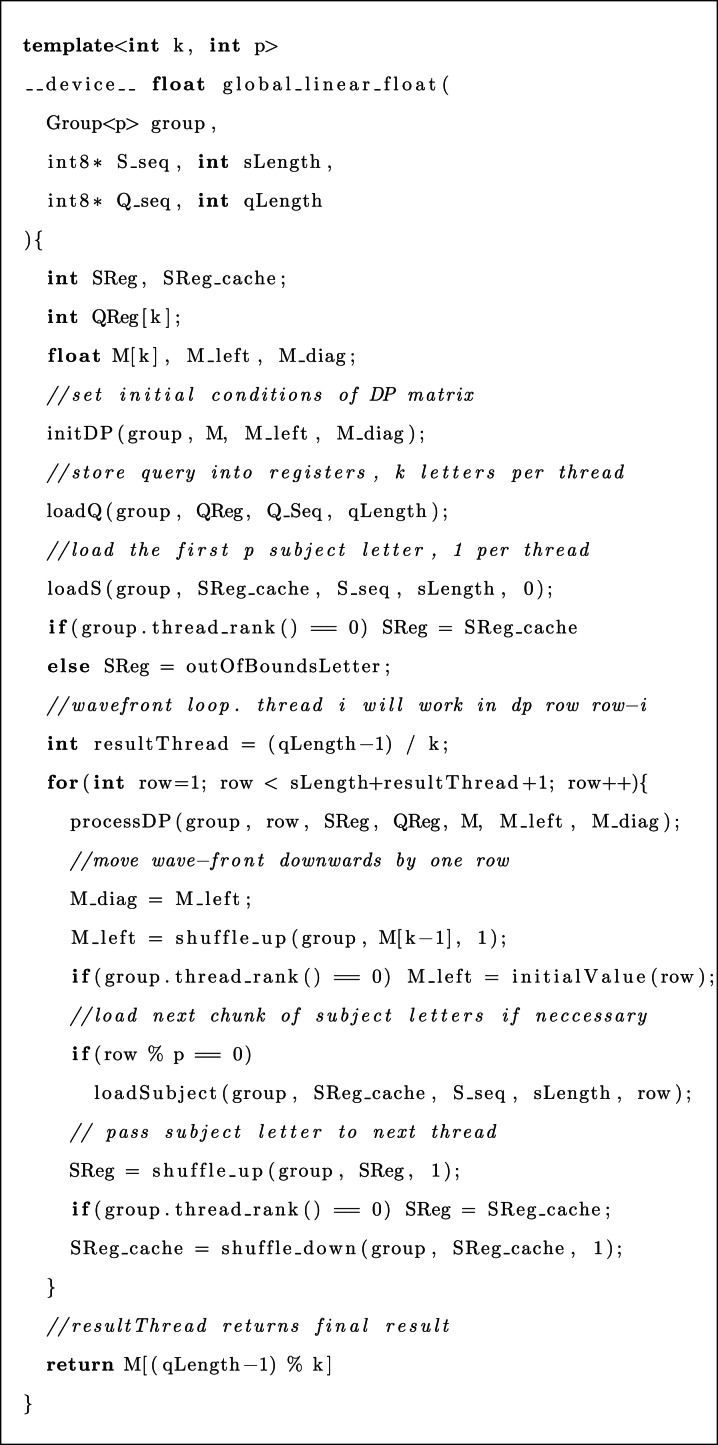

### Kernel variants

All computational kernels provided by our library follow the general code structure presented in Listing 1. We use optimized functions tailored towards the computation of global alignment, local alignment, and semi-global alignment, with different penalty schemes and datatypes. In the following, we will describe implementation details for each algorithm. We begin with observations which hold true for all kernels.

For each algorithm type, we provide a so called *AlignmentState* which represents the wavefront. It encapsulates the DP matrix registers and contains the logic to initialize and compute the DP matrix cells. This allows for a simple switch between optimized implementations for linear gap penalty and affine gap penalty, as the gap penalty scheme is independent from the movement of the wavefront within the DP matrix.

The efficiency of Accelign relies on storing and computing DP cells in thread-local registers. However, thread-local arrays are typically only materialized in registers if the compiler can compute all accessing indices at compile-time. We achieve this by manual unrolling or automatic loop unrolling using #pragma unroll.

The DP cells can be computed using different datatypes. Our library supports scalar types like 32-bit float and int, as well as vector types with two components like packed 16-bit types half2 and short2. In addition, we provide a specialized kernel for one-to-all local alignments that utilizes packed 8-bit integers, uint8x4, on Blackwell. Our mathematical operations are specialized for each supported datatype. For example, the operation max(a+b,c) which is common to alignment computations is implemented as written for floats, but uses a specialized function __viaddmax_s32(a,b,c) for integers, which maps to a single hardware DPX instruction on supporting GPU architectures like NVIDIA Hopper or Blackwell GPUs.

We do not provide specialized kernels for DNA or Proteins per se. Instead, our alignment kernels can simply be specialized for a specific alphabet size $|\Sigma |$ which is passed as a template argument. All input characters must be encoded in the range $[0, |\Sigma |-1]$. Our library does not pose additional restrictions to the encoding. Internally, the alphabet size $|\Sigma '| = |\Sigma |+1$ is used. It contains an additional element representing an out-of-bounds character, which is returned by out-of-bounds accesses to input sequences. Out-of-bounds accesses occur when the wavefront is not fully contained in the DP matrix. When half2 or short2 is used, each CG computes two independent alignments in the upper and lower 16 bits, respectively. Internally, the corresponding two query sequences and the two subject sequences will be zipped together producing a new sequence, respectively, encoded in the range $[0, |\Sigma '|^2-1]$.

The resulting new sequence has a length equal to the longer of the two zipped sequences. Therefore, two adjacent sequences in an alignment batch should have the same length or similar length to avoid load imbalances during alignment computation. Following the same principles, four alignments per CG are computed when utilizing the uint8x4 integer instructions, with four sequences being zipped together.

Next, we present details specific to each supported alignment algorithm.

#### Global alignment

The optimal score of a global pairwise alignment is always stored in the bottom-right cell of the DP matrix. In our wavefront, this cell can in theory be covered by any thread, but more importantly it appears at position M[(qLength-1)%k], which is an array index not known at compile-time. To ensure that the compiler can still place the array in registers, we explicitly copy the array to local memory after computation is complete to access it via dynamic indexing without restrictions. A different challenge regarding the output arises when using the packed 16-bit types. In general, input sequences can be of different length. The global alignment score of any sequence must be output as soon as its cell has been computed. Otherwise, the value may be overwritten in the next row. Therefore, we need to split the alignment loop into two parts when dealing with half2 or short2: First, compute all rows required for the shorter of the two sequences, and output the respective score. Then, continue until the longer sequence is fully processed.

#### Local alignment

An optimal local alignment score computation returns the maximum observed value. During wavefront computation, each thread thus keeps track of its observed maximum score. After the DP matrix is fully processed, a group-wide parallel reduction is performed to find the total maximum across the group. It is then returned. Note that for global alignments the computation of out-of-bounds DP cells cannot affect the final score as the thread which holds the result is fixed for a given alignment calculation. However, this may not be the case for a local alignment. The out-of-bounds computation could change the per-thread observed maximum, and the total maximum could be located in any thread. To avoid this issue, we use a substitution score $\le 0$ for any out-of-bounds positions. Thus, the per-thread observed maximum can never change by out-of-bounds DP cell calculations. This approach is also beneficial when using half2 or short2. Since computation can arbitrarily extend beyond the DP matrix without changing the final result, the alignment loop does not need to be split into two parts. A single loop with maximum number of DP rows is sufficient.

Compared to a global alignment, a local alignment performs an additional clamping to 0. Although CUDA provides specialized (DPX) functions for int and short2 to compute $\max (\max (a+b,c),0)$, it is only hardware-accelerated since the Hopper architecture, and no such function exists for float and half2. However, the operation $\max (a+b,0)$ can be implemented for half2 using a fused multiply-add with integrated relu, __hfma2_relu(a,half2(1,1),b), which maps to a single hardware instruction on all GPU architectures since Ampere. The local alignment recurrence can be reformulated using $\max (a+b,0)$ which allows the usage of the specialized function for half2 which saves one instruction in the inner loop. For example, with linear gap penalty, the instruction sequence

$$\begin{aligned} \texttt {relu(max(add(max(left, up), gap), add(diag,}\, \sigma (s_i,q_j))\texttt {)} \end{aligned}$$

can be rewritten as

$$\begin{aligned} \texttt {max(add}\_\texttt {relu(max(left, up), gap), add}\_\texttt {relu(diag,}\, \sigma (s_i,q_j))\texttt {)}\end{aligned}$$

This is based on the observation that if the score produced by one direction is negative, it can never become the new maximum as the maximum is at least 0. Thus, negative values can be clamped to 0. Then, since all the directions now produce a non-negative score, the final relu operation can be omitted. We also apply a similar code transformation for affine gap penalty.

Our uint8x4 integer implementation uses unsigned integers, enabling scores between 0 and 255. Saturated arithmetic is employed to avoid unsigned wrap-around. To allow negative substitution scores, we first identify the absolute value of the smallest non-positive entry of the substitution matrix. Subsequently, this value, called bias, is added to all entries of the substitution matrix to make them non-negative. This bias is subtracted from each cell during alignment computation, resulting in the instruction sequence

$$\begin{aligned} \texttt {max(sub(max(left, up), gap}\_\texttt {abs), sub(add(diag,}\, \sigma ^*(s_i,q_j)\texttt {)}, \texttt {bias))}\end{aligned}$$

for a linear gap penalty, where $\sigma ^*(s_i,q_j) = \sigma (s_i,q_j) + bias$.

#### Semi-global alignment

Computing a semi-global alignment requires tracking the maximum in the last column of the DP matrix. As explained previously, the thread ID and the per-thread column ID for the last column of the DP matrix depends on the query length and is not know at compile-time. To avoid conditional branches in the inner loop, we unconditionally track the maximum of all columns at the cost of higher register pressure. The wavefront loop needs to be split into multiple parts. Part A includes all iterations where the wavefront covers the initialization row of the DP matrix. Part C includes all iterations covering the last row of the DP matrix. In between, there is part B which contains all other iterations. Note that for short sequences part B can be empty, and parts A and C may overlap. Part A is necessary to explicitly initialize the first row of the DP matrix to 0 for threads other than the first thread. In contrast, local alignment does not require explicit initialization because 0 is implicitly generated via the relu operations. Part C enables maximum tracking within the last row. Similar to local alignment, this produces a per-thread local maximum which is transformed into a total maximum via group-wide parallel reduction. After the computations are complete, the thread which covers the last DP matrix column computes the maximum between the total row maximum and the maximum observed in the last DP matrix column to produce the final result.

For global alignment with packed 16-bit types, we split the wavefront loop into two part to account for the potentially different matrix sizes of the two sequences. This is further complicated for semi-global alignment, leading to five parts. Part A for the initialization, part C and part E for tracking the last row in the two DP matrices, and parts B and D for the remaining iterations.

### PSSM and substitution matrix lookups

Common to all of our alignment variations is the need to compare a position in the subject with a position in the query, producing a substitution score $\sigma (s_i,q_j)$. Such substitution scores are commonly stored in memory as substitution matrix or PSSM. Note that simple scoring approaches could also compare the two letters explicitly to produce a *matchscore* or *mismatchscore*.

**Fig. 3:**
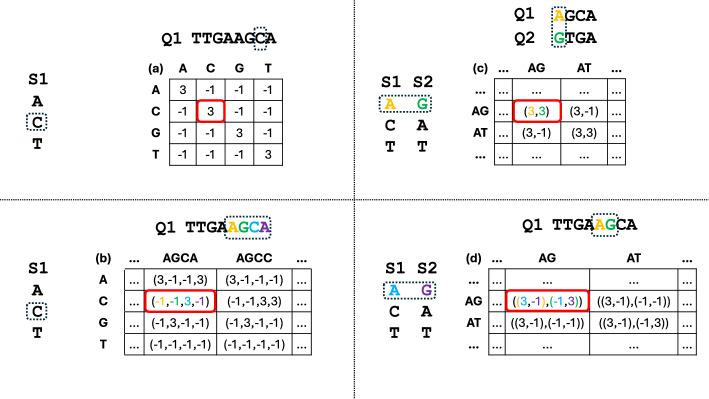
Substitution scores of substrings in dashed boxes are given by red boxes within a substitution matrix. Numbers and letters are colored to indicate corresponding scores. The specified input substitution matrix will be converted into one of many internal layouts. These include: (**a**) Traditional layout. (**b**) Substitution matrix for an alignment using scalar (e.g. float) computations. All combinations of four letters are stored in one dimension to load substitution scores of four consecutive letters in one sequence. (**c**) Layout for vector (e.g. half2) computations. Two zipped sequences (S1,S2) are aligned against two zipped sequences (Q1,Q2). Substitution matrix stores the element-wise comparison result. (**d**) Layout for vector (e.g. half2) computations. Two zipped sequences (S1,S2) are aligned against one sequence (Q1). A pair of zipped letters is compared against two consecutive letters of the single sequence

Our alignments support both a substitution matrix and a PSSM. The simple case of explicit comparison could be modeled by a substitution matrix which stores *matchscore* on its main diagonal, and *mismatchscore* otherwise (see Fig. 3(a)). Note that for a given query and substitution matrix it is trivial to compute a corresponding PSSM.

In our implementation we use a level of abstraction which allows for simple integration into the main algorithm. The substitution scores are returned by a so called *SubstitutionScoreProvider*. It provides functions which take $s_i$ as input and return the substitution score for one or multiple columns covered by a thread. We implemented different substitution score providers for PSSM and substitution matrix, and for different score datatypes.

When calling an alignment kernel, the scoring matrix or PSSM is initially stored in (high latency) global memory. As a first step before computing any alignments, the substitution scores are copied into faster shared memory which can be accessed by all threads within a thread block. Current generations of CUDA-enabled GPUs typically provide at least 99KB of shared memory per thread block. To efficiently access shared memory, one needs to use an appropriate access pattern. Shared memory is accessed by a full warp; i.e., 32 consecutive threads. It is organized into 32 4-byte memory banks which can operate in parallel. Each shared memory address is served by a specific fixed memory bank. The *i*-th four-byte value in the shared memory address space corresponds to bank $b=i \mod 32$. A warp-wide access to shared memory is decomposed by hardware into 128-byte-wide transactions. A shared memory bank conflict occurs if multiple different addresses which map to the same memory bank are part of the same transaction. As the memory bank cannot serve multiple different addresses at the same time, the transaction needs to be replayed as often as required to serve all addresses of the transaction. If possible, one should minimize the number of shared memory bank conflicts as they reduce the memory throughput.

After covering the fundamentals, we will now explain the implementations of the different score providers in more detail.

#### Substitution matrix

Accelign allows the use of a substitution matrix for all alignment application scenarios. It is passed as an integer matrix of size $|\Sigma | \times |\Sigma |$. Internally, we can choose between multiple matrix shapes with respect to

- matrix dimensions,
- layout in shared memory, and
- data type.

The specific choice depends on the use case and is a trade-off between

- shared memory access speed,
- shared memory size, and
- executed instruction count.

First, consider the case where a scalar score type is used for DP cell computations and the matrix shape is $|\Sigma '| \times |\Sigma '|$ storing the same scalar type.

Since all input sequences are properly encoded as integers, we can use those integers as index into the substitution matrix to return the corresponding substitution value. Note that this requires to keep the query letters in registers to avoid reloading them in each iteration of the wavefront loop. Considering a whole CG, the access pattern for the substitution matrix is random access, as each thread within the group effectively operates with different subject/query value pairs. In particular, this implies random access to shared memory within a warp. Thus, we are susceptible to bank conflicts as the memory bank requested by each thread is effectively random. However, in the case of DNA alignments with $\Sigma = \{A,C,G,T,N\}$ this is not a problem. The substitution matrix has a memory size of just 144 bytes (when using float or int), where the last $6\cdot 4=24$ bytes are only ever accessed for out-of-bounds subject letters. This leaves 30 elements to be accessed frequently, each assigned to a different bank. Thus, for the majority of accesses there will be no bank conflicts. For larger alphabets such as those used for proteins, however, this no longer holds true.

Note that one shared memory lookup per DP cell is performed in the case described above. This could be reduced by using wider memory loads, loading multiple substitution scores per instruction. Yet, due to the random access pattern it is unclear if the additionally loaded elements are necessary for subsequent DP calculations. However, by increasing the size of the substitution matrix in shared memory it is possible to utilize wider memory loads without loading unused data. This can be achieved by using a row and/or column for each possible combination of two or four elements of the alphabet (see Fig. 3(b)). The substitution scores of multiple consecutive sequence positions can then be obtained by a single look-up using the respective combination of letters as the index into the inflated substitution matrix. This concept is particularly important for DP calculations using vector types like half2 which require two substitution scores for the different alignments in each vector lane. Examples of different matrix layouts with their use-case are shown in Figure 3.

In addition to the different matrix shapes, a second degree of freedom is the choice of a substitution score datatype. To reduce the amount of shared memory used by the substitution matrix, one could consider using a narrower element datatype such as 16-bit floats instead of 32-bit floats. This can prove beneficial for performance even if it introduces an additional instruction to convert from the narrower type to the type used for DP cell calculation. For example, we observed a case where the performance was limited by an oversubscribed memory input/output (MIO) queue. The optimal matrix layout for this case stored all combinations of 4 letters and used 16-bit floats. While this did not reduce the number of instructions (4 32-bit loads vs 1 64-bit load + 4 conversion instructions), it shifted work to a lesser utilized hardware pipeline which in turn led to increased performance.

#### PSSM

A PSSM typically requires significantly more memory than a substitution matrix because its size depends not only on the alphabet size, but also on the query length, or rather $p \cdot k$, the maximum number of columns which fit the CG. Due to the potentially high shared memory requirements when using a PSSM, we only support PSSM in the case of one-to-many alignments which requires to store only a single PSSM in shared memory. From a performance perspective a PSSM allows for the best possible shared memory access pattern. The reason is two-fold:

1. Each thread in a group accesses a fixed distinct set of *k* consecutive columns of the PSSM; i.e., thread *i* will only access columns $[i\cdot k, (i+1)\cdot k)$. Therefore, it is possible to reorder the PSSM columns in memory to enable bank-conflict-free accesses.
2. Since a thread requires multiple consecutive elements within the same PSSM row, 16-byte-wide memory load instructions can be used to reduce instruction count in the innermost loop. Those fetch four required 32-bit substitution scores in a single instruction.

Additionally, in contrast to substitution matrix, since the mapping of per-thread DP matrix columns to shared memory PSSM columns is fixed, the actual query letters are not longer required which reduces register pressure.

One particular challenge is to provide bank-conflict-free accesses for kernel configurations where the total amount of bytes loaded per CG in one instruction is less than the shared memory transaction size of 128 bytes. This case arises for example with a group size of 4 and 16-byte loads. Then, the loads of the next CG, which will access the same PSSM columns, but from potentially different rows, are included in the same 128-byte transaction. This can lead to a bank conflict. Our solution is to replicate the PSSM in shared memory as often as needed to ensure that groups which are part of the same transaction access distinct memory banks, leading to conflict-free access. In the described example with four threads and 16-byte loads, we utilize two PSSMs which are interleaved such that the *g*-th group only accesses memory banks $0-15$ when *g* is even, and only accesses banks $16-31$ when *g* is odd.

**Fig. 4:**
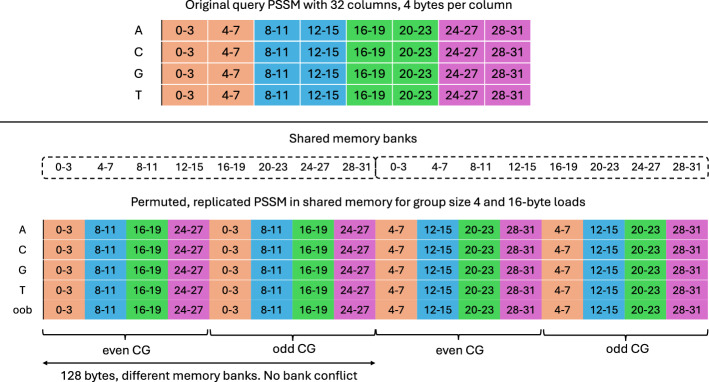
A PSSM with 32 columns should be aligned by CGs with 4 threads, and 8 columns per thread, i.e. $p=4,k=8$. Different colors indicate different threads within a CG. Since threads in a CG execute concurrently, PSSM columns which are accessed at the same time should be stored consecutively which requires a permutation of columns. By interleaving two copies of a permuted PSSM, two CGs can be served by a single conflict-free 128-byte transaction which uses distinct memory banks

Figure 4 provides an example of a conflict-free PSSM layout in shared memory.

#### Memory usage and its limitations

Conceptually, our library supports any alphabet size. However, in practice the alphabet size is limited by the available shared memory to hold the PSSM or substitution matrix. Especially for the half2 or short2 types, where memory grows at least quadratically with alphabet size a limit is quickly reached. To enable the usage of those types with alphabet sizes typical for bioinformatics applications, e.g. protein alphabet, we have also implemented low-memory score providers. Here, the value type stored in the data structure are only half or short instead of half2 and short2. Additionally, we no longer use the zipped alphabet to access the data. Instead we perform a separate lookup for each of the two sequences and pack the two values into a half2 or short2 for calculation. Using this low-memory approach the shared memory size only grows linearly with alphabet size for half2 and at short2 the cost of additional instructions

for loading and interleaving data. Similarly, four lookups are required with the uint8x4 integer implementation.

With 99 kilobyte of shared memory per block and $|\Sigma |=25$, the low memory variant for half2 and short2 is necessary for both the PSSM and the one-to-one substitution matrix for most matrix tile sizes. The maximum alphabet size for which the low-memory half2 PSSM variant is able to process a query sequence of length 1024 with 99 kilobytes shared memory is 48 when using 2-byte storage types. A low-memory half2 substitution matrix can be used with alphabet sizes up to at least 128.

Note that the simplest approach to bypass shared memory size limitations would be to keep all data in global memory. While functional, this would be prohibitively slow. Our library therefore does not offer this option.

### Finding start and end positions of alignments

At present time, Accelign is designed to compute alignment scores, but cannot compute the full traceback paths. However, for local alignment and semi-global alignment we provide additional kernels that are able to identify the end coordinates of the alignment path within the DP matrix, and optionally computing the start coordinates as well. Functionality of computing start/end positions is limited to alignments with substitution matrix and datatypes float and int. PSSM and packed 16-bit types are currently not supported.

To solve the task of finding the end coordinate the algorithms proceed as usual, with the addition of keeping track of the coordinate of the current observed maximum score in the DP matrix for local alignment (or in the last row and last column in the case of semi-global alignment). While updating the observed maximum can be done in a single instruction, explicit conditionals are required to determine if the observed maximum changed, and if yes, to update the corresponding cell coordinates. This adds additional instructions in the innermost loop which reduces the performance compared to score-only alignments.

Our approach to find the start coordinates is the computation of an optimal alignment of the reversed input sequences. Let *S* and *Q* be the alignment input sequences with $|S| = n$ and $|Q| = m$. We first use the aforementioned approach to find the optimal alignment score *O* and its DP cell coordinates $(e_s, e_q)$ which corresponds to the end position of the alignment. This gives us the information that the optimal alignment is produced by substrings $S[1:e_s]$ and $Q[1:e_q]$. Next, we compute an alignment with end position of the reversed substrings $S'=rev(S[1:e_s])$ and $Q'=rev(Q[1:e_q])$. This gives DP cell coordinates $(e_s', e_q')$ of the end of the reverse alignment, which can be converted into start coordinates $(b_s, b_q)$ of the original forward alignment. Using these start and end coordinates, external libraries could be used to compute a global alignment between $S[b_s:e_s]$ and $Q[b_q:e_q]$ to obtain a traceback.

As an optimization to our approach, we use the already computed alignment score from the forward alignment to short-cut the reverse substring alignment. As soon as the optimal alignment score is observed in the DP matrix, we can conclude that the end has been found as there cannot be a better score, and terminate.

### Partitioning for long sequences

Our kernels can be configured to support different query lengths via parameters *p* and *k*. However, *p* and *k* cannot be increased indefinitely due to hardware limitations. First, warp shuffles can only be used for $p \le 32$. Second, the *k* DP matrix cells need to be kept in per-thread registers, whose number is limited. Excessive register spilling to slow global memory will hinder performance. Our solution to support queries of length $l > p \cdot k$ is matrix tiling. We define the *tile size* as $z = p \cdot k$. Then the DP matrix can be partitioned into $\lceil l/z \rceil$ many matrix tiles with up to *z* columns per tile. A single CG then processes the matrix tiles in order. When the CG processes any matrix tile which is not the last tile, it writes the computed DP cells of the last column of the tile to temporary storage in global memory. This enables processing of the successor tile. The saved DP cells are required to serve the diagonal and left dependency for the first column the successor tile, and are loaded on demand.

The size of the required temporary storage per CG depends on the maximum subject length $\max (l_s)$ to be processed, the group size *p*, and the number of bytes to be written per DP cell. Specifically, our current implementations requires $(\max (l_s) + p) * cell size$ bytes per CG. For alignments with linear gap penalty, 4-byte elements are used. The temporary storage for alignments with affine gap penalty requires 8 bytes per cell.

Note that in principle this approach requires separate temporary storage for each alignment, which can easily exceed the available GPU memory for larger batches / longer queries. However, the maximum number of CGs which can execute concurrently on the GPU is limited by finite hardware resources on an SM, such as available shared memory or registers. Thus, it is sufficient to launch the minimum number of CGs to saturate the GPU resources, and have each CG compute multiple alignments consecutively, re-using the temporary storage of the CG. Accelign uses a grid-strided for-loop to enable processing of multiple alignments per CG in a round-robin manner.

### Accuracy of data types for computing alignment scores

By using SIMD-like instructions to operate on two 16-bit quantities packed in a 32-bit value, it is theoretically possible to achieve up to two-fold speedup. However, using 16-bit numbers for sequence alignment can have draw-backs in terms of accuracy. Regardless of the used data type, only integer scores are represented. The 16-bit half datatype can represent all exact integer values *x* where $-2048 \le x \le 2048$. For 16-bit shorts the range is $-32768 \le x \le 32767$. Integer values outside of these ranges are rounded towards zero for half. For example, the value 2049 is rounded to 2048. In the case of shorts, no values outside the given range are representable, and signed integer overflow/underflow may occur during calculations, which is undefined behavior in the C++ standard.

Accelign does not check if a value cannot be represented during computations. Such checks would cancel out the performance advantage of 16-bit types due to increased instruction count. We recommend using the traditional 32-bit types instead if a wider range of values needs to be supported. Nevertheless, for local alignments a simple post-processing step can identify a score as exact. Let *x* be the final 16-bit alignment score, and *b* the largest entry in the substitution matrix. For all cases where $x < 2048$ (half2) or $x+b \le 32767$ (short2), *x* is the exact result.

When employing the uint8x4 implementation, alignment scores less than $255 - bias$ are exact.

## Results

**Table 2:** Summary of the hardware specifications of the systems used in our evaluation

	CPU			GPU				
	CPU Model (Architecture)	Cores (Threads)	RAM	GPU Model (Architecture)	SMs	VRAM	ShMem	DPX
S1	EPYC 7713P (Zen 3, Milan)	64 (128)	512GB	RTX PRO 6000 (Blackwell, 2x)	188	96GB GDDR7	100KB	✓
S2	Xeon Gold 6548Y+ (Sapphire Rapids, 2x)	2$\times$32 (128)	1TB	L40S (Ada, 2x)	142	48GB GDDR6	100KB	✕
S3	EPYC 7713P (Zen 3, Milan)	64 (128)	512GB	A100 (Ampere)	108	80GB HBM2e	164KB	✕
S4	ARM Neoverse-V2 (Grace)	72 (72)	480GB	GH200 (Hopper)	132	96GB HBM3e	228KB	✓

We evaluated the performance of Accelign on the four machines S1-S4 with specifications listed in Table 2.

The primary system used for our evaluation are S1 and S4. Tother systems are used for comparison between different GPU generations on a synthetic benchmark. CUDA 13.0 and gcc 9.3.0 are used as GPU and host compiler, respectively. GPU code using uint8x4 was compiled with CUDA 13.2.

As a preliminary step, on each system we conducted a grid search over a subset of possible alignment configuration parameters for alphabet sizes 5 (DNA/RNA) and 25 (Protein). The results were used to derive a set of GPU-specific matrix tile sizes, thread configurations, and PSSM/substitution matrix configurations for best performance.

Next, based on the obtained configurations we developed a high-level convenience API on top of our low-level kernel-based API. The uint8x4 integer local alignment is currently only available using the low-level API. Given a batch of sequence pairs to align, the high-level API is able to select a suitable alignment configuration based on the maximum query length observed within the batch. For queries with a length $\le 512$ a single matrix tile is used whose size is the best fit to the query length. For longer queries, we switch to multi-tile processing with a tile size of up to 1024. The selected tile size requires the least number of tiles and performs the fewest out-of-bounds computations. Substitution scores are restricted to the range $[-15,15]$ since some configurations make use of the 8-bit floating point type __nv_fp8_e4m3. This still allows us to use the common BLOSUM substitution matrices for protein alignments. It is possible to prevent usage of this type, which increases the supported range to $[-2048,2048]$.

In the following, we demonstrate the performance of our library in different synthetic and real-world case studies. For one-to-all alignments the PSSM approach is used. In the case of one-to-one alignments a substitution matrix is used instead. Unless stated otherwise, the high-level API is used.

### Performance scaling with sequence length

In this synthetic benchmark we processed a batch of simulated sequences of equal lengths. Sequence length was varied between 32 and 65536 in steps of powers-of-two.

Note that these benchmarks are only intended to highlight performance and ignore inaccuracies for longer sequences caused by 16-bit computations. The batchsize was limited to 4 gigabytes worth of data, and $2^{26}$ sequences. We gathered performance data for all of our supported algorithms, alphabet sizes 5 and 25, and different data types used for computation.

**Fig. 5:**
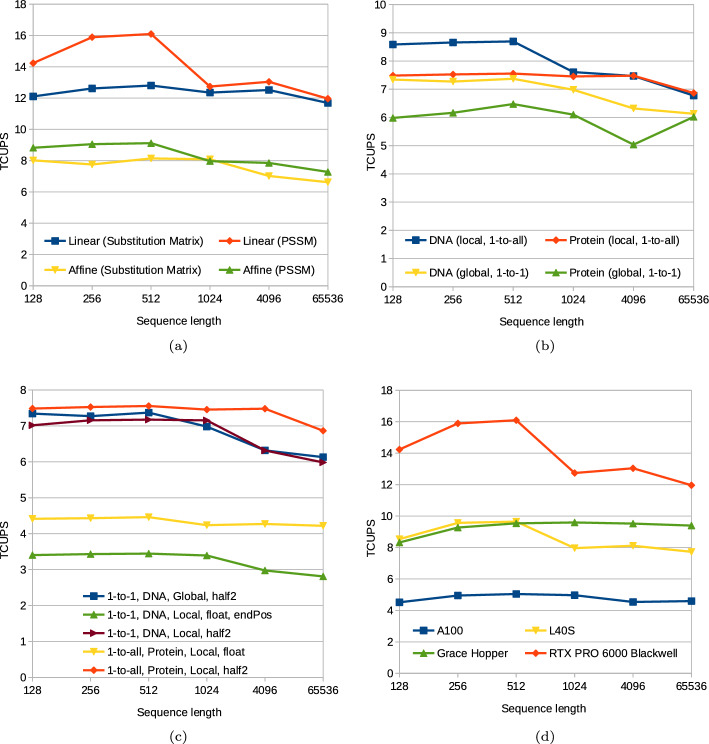
(**a**) Performance comparison between substitution matrix and PSSM using a single GPU on S1. A global alignment of DNA was used with 16-bit half2 computations. (**b**) Performance comparison between alphabets for DNA/RNA and proteins using 16-bit half2 computations with affine gap penalty using a single GPU on S1. (**c**) Synthetic benchmark of different kernels with affine gap penalty used in our real-world case studies on a single GPU of S1. (**d**) Performance comparison of different GPU generations computing a 1-to-all DNA global alignment with linear gap penalty, half2 computations, and PSSM

We measure performance in terms of matrix cell updates that are performed per second; for example, TCUPS (Trillions of Cell Updates Per Second) as

6 $$\begin{aligned} \textrm{TCUPS} = \frac{\sum _i m_i \times n_i}{t \times 10^{12}}, \end{aligned}$$

where *t* is the runtime in seconds and $m_i$ and $n_i$ are the lengths of the aligned sequences. Here, we only give a summary of our findings in Figure 5. The full set of benchmark results can be found in the Supplementary File.

First, we motivate the choice of PSSM for one-to-all alignments by taking a look at the performance differences between PSSM and substitution matrix. Substitution matrix performance was measured using the kernel-level API. Figure 5a shows the achieved performance on single GPU of S1 across the different sequence lengths for both approaches for a global alignment of DNA sequences using half2. Calculating a global alignment with linear gap penalty requires the fewest computations per DP cell. Any inefficiencies in shared memory accesses and instruction count will thus be noticeable. Indeed, for short queries with a length up to 512 the PSSM variant is up to $29\%$ faster. For long queries, performance is impacted by global memory accesses due to matrix tiling, hiding the benefits of PSSM. Still, a speedup of $5\%$ can be observed. On the other hand, alignments with an affine gap penalty come with a greater compute-to-memory-access-ratio. Here, using a PSSM improves the performance by up to $16\%$ $(11\%)$ on short (long) queries.

Next, Figure 5b shows the performance differences between the two alphabet sizes when using half2 and affine gap penalties. In this case, alignment kernels are more efficient when applied to DNA sequences. This is because of the aforementioned challenges in shared memory usage. Additional instructions are required for the protein alphabet with low-memory variants of PSSM and substitution matrix. When 32-bit floats are used instead (not present in the figure), one-to-all alignments show similar performance on both alphabets because the same PSSM layouts and storage types can be used. On the other hand, one-to-one alignments are faster on the smaller alphabet because a substitution matrix layout which supports a wider memory access size can be employed.

In our real-world case studies, a number of different alignment algorithms were used. Figure 5c highlights the corresponding performance. The half2 version of a one-to-all local affine alignment on proteins is around $70\%$ faster than the float version. For one-to-one alignments, computing the alignment end position in addition to the optimal score costs up to $25\%$ performance.

Last, Figure 5d provides a performance comparison between the latest Blackwell generation and prior CUDA-enabled GPU architectures. We have chosen a DNA one-to-all linear global alignment with half2 because it is the kernel which achieves the highest TCUPS across all tested GPUs. On this kernel, RTX PRO 6000 Blackwell is between 2.6x and 3.4x faster than A100, and between 1.5x and 1.9x faster than L40S, showing that performance increases with newer GPU generations.

In addition, we compared the performance of our fastest kernel on RTX PRO 6000 Blackwell against the *theoretical peak performance* (TPP) for the arithmetic instruction sequence of a global alignment with linear gap penalty, i.e. $\texttt {max(add(max(left, up), gap), add(diag, substscore))}$.

The theoretical peak performance of the GPU hardware is calculated as:

7 $$\begin{aligned} \textrm{TPP} = \frac{\mathrm {\#SMs} \times \mathrm {Throughput\_per\_instruction} \times \textrm{Clock} }{\mathrm {Cycles\_per\_cell\_update}} \end{aligned}$$

The utilized Blackwell GPU has a documented throughput of 64 results per clock cycle per SM for half2 addition (HADD2),^1^ and we have measured the same throughput for half2 maximum computation (HMNMX2) via microbenchmarks. HADD2 instructions execute in the FMA pipe whereas HMNMX2 instructions execute in the ALU pipe. This gives the opportunity to process independent addition and maximum simultaneously, and would allow for the concurrent computation of max(left, up) and add(diag, substscore) resulting in three clock cycles to compute two DP cells. With a boost clock of 2.617 GHz and 188 SMs, this yields a TPP of $(188 \times 64 \times 2.617) / 1.5 = 20.99$ TCUPS.

Provided the synthetic benchmark in Figure 5d on a single RTX PRO 6000 achieves a performance of up to 16.1 TCUPS, our approach is able to achieve an efficiency of 77 %. This demonstrates that our in-register computations in conjunction with efficient warp shuffles and shared memory accesses result in a compute-bound alignment implementation.

### Case studies

In the following case studies, we demonstrate the benefits of using our GPU-accelerated alignments with Accelign in three problem settings: DNA short-read alignment, Protein database searches, and HIV short-read mapping.

### Short-read alignment

We used Accelign to create a simple short read alignment tool. Given two fasta files with DNA sequences as input, it computes the global affine alignment score between the $i-$th sequence of each file. This task maps to the one-to-one case of our library with alphabet size 5. The program uses a three-stage pipeline with a dedicated CPU thread per stage. The first stage generates batches of sequences to be aligned. As part of a larger framework, this stage could involve other (pre-)processing steps. In our program, we simply parse the input files. The second stage receives such a batch from the first stage and aligns the contained sequences. This could be achieved by calling a CPU library or GPU library. In the case of GPU libraries, the typical workflow includes transferring the sequence data to the GPU, and subsequently executing a GPU kernel for alignment. Additional set up may be performed on the CPU. After the alignment kernel completes, the alignment scores are transferred back to the CPU. The last pipeline stage receives a batch of sequences and their corresponding alignment scores for further processing. Our benchmark tool creates an output file from the previously calculated alignment scores.

GPU alignment libraries often expect a list of CPU-side DNA strings as input and hide the required data transfers to the GPU from the user. While such an interface is simple to use, it has disadvantages in regards to performance. First, all sequence strings internally need to be copied into a contiguous staging buffer for efficient data transfer. Second, if the sequences were already present in GPU memory, they would first need to be copied to the CPU and transformed into strings before the alignment library could be called (copying them back to the GPU). Third, maintaining a large collection of separate strings may lead to memory fragmentation and poor data locality. In contrast, Accelign only provides the core alignment functions which expect the input data to already be present in GPU memory. Data transfer is explicitly managed by the user. Importantly, this allows us to accept the alignment input data in a contiguous data layout, which avoids creating an intermediate copy of the data. Thus, our first stage provides the sequence data in two formats; a list of separate strings for other APIs, and a contiguous buffer of sequence data for our API.

With above alignment framework set up, we measured the alignment performance of Accelign and compared it against other libraries. For this, we implemented the second pipeline stage for each library. Runtimes are only measured for this stage. We compared our library to the GPU-based implementations ADEPT, GASAL2, and WFA-GPU. In addition, we used Parasail 2.6.2, Seqan 3.4.0, BSAlign 1.2.1, KSW2, EdLib 1.2.7 , WFA2 2.3.6, and A*PA2, as CPU-based alignment libraries.

Note that we are only interested in the global alignment score, but not the traceback. For all implementations that support tracebacks, traceback computation was disabled for this benchmark, if possible, by either passing the corresponding options to the aligner, or using the appropriate alignment function. Furthermore, we disabled banded alignments, if possible, to focus on the performance of computing the full DP matrix. Note that in real use-cases banding strategies are preferred to avoid the exhaustive search, thus improving performance. In particular, the following library settings in regards to banding and traceback were used: CIGAR::NO was passed to Adept; neither traceback nor banding could be disabled for A*PA2 (function astarpa2_simple); Bandwidth=0 and cigar=NULL were passed to bsalign; Edlib used automatic banding and EDLIB_TASK_DISTANCE; GASAL2 was run without computation of start positions; KSW2 used KSW_EZ_SCORE_ONLY with both banding and z-drop disabled; Parasail functions without banding and traceback were used; Seqan3 was used with both banding and traceback disabled; AlignmentScope::Score was passed to WFA2 and no banding was enabled; Last, we set compute_cigar=false and band=BAND_NONE for WFA-GPU. The CPU benchmarks were performed on system S1 with 128 threads since these typically scale with the number of logical cores. System S4 was used for GPU benchmarks as it provides the fastest CPU-GPU interconnect, eliminating bottlenecks caused by data transfers.

A simple scoring scheme was used with gap opening $= -3$, gap extension and mismatch set to $-1$, and match score $=0$. The match score was set to 0 because it is a precondition for WFA-GPU and WFA2 to work. Note that EdLib and A*PA2 are only able to use the very restricted edit distance as scoring scheme.

To generate the input data for our benchmark, we used BWA [36] to map Illumina human sequencing reads (accession number SRR1766553^2^) against reference HG002, and extracted the corresponding genome coordinates. The benchmark then attempts to align each read to its determined genomic subsection. In total, our benchmark dataset contains 10 million reads of length 148. The genomic subsections have minimum length of 103, a maximum length of 209, with an average of 148. The number of DP cells to be computed is approximately $219 \cdot 10^9$.

Table 3 presents the runtimes for pipeline stage two. Since it is difficult to measure GPU-side activity accurately when transfers and GPU kernel launches are hidden behind the API, we used the NVIDIA Nsight Systems profiler with NVTX ranges to obtain durations of GPU activities as well as the total time spent on processing the sequence batches.

**Table 3:** Runtimes (in milliseconds) and TCUPS for the second pipeline stage on system S4. Individual runtime contributions are only given for the (no overlap) case. Total runtime includes contributions not shown in the table. Accelign uses half2 computations. Alignment TCUPS indicate raw kernel performance (computed from the alignment kernel runtime), whereas best total TCUPS is computed from the best total runtime

	GPU libraries				CPU libraries						
	Acce- lign	GASAL2	ADEPT	WFA GPU	Parasail	Seqan	BSAlign	EdLib	KSW2	WFA2	A*PA2
Buffer preparation	–	374.9	534.0	416.8	–	–	–	–	–	–	–
CPU-GPU transfer	8.6	9.3	8.4	9.5	–	-	–	–	-	–	–
Alignment kernels	57.0	106.9	1,831	48.7	–	–	–	–	–	–	–
Other kernels	5.8	1.4	–	45.2	–	–	–	–	–	–	–
CPU alignment	–	–	–	22	13,796	2,089	3,854	672	2,982	126	828
Total runtime											
No overlap	71.9	499.1	2,388	997.3	13,796	2,089	3,854	672	2,982	126	828
With overlap	62.4	379.3	–	910.2	–	–	–	–	–	–	
TCUPS											
Alignment	3.84	2.05	0.12	3.10	0.02	0.10	0.06	0.33	0.07	1.74	0.26
Best total	3.51	0.57	0.09	0.24	0.02	0.10	0.06	0.33	0.07	1.74	0.26

Accelign achieves the fastest runtime for alignments which corresponds to 3.8 TCUPS. Compared to a synthetic peak performance benchmark, this is around $20\%$ less than the maximum observed TCUPS. This can be explained by inefficiencies due to non-equal sequence lengths in the real-world data as opposed to the synthetic case. WFA-GPU has a fast alignment kernel as well but is unable to compute all alignment scores on the GPU due to algorithmic limitations. It needs to fall back to CPU-based alignments for those cases which reduces performance. ADEPT is outclassed by all other tested GPU libraries, and might be slower than fast CPU aligners on capable hardware. WFA2 is noticably the fastest of the tested CPU libraries. Since it also avoids the overheads inherent to most of the GPU libraries, it is the second fastest of all tested libraries in terms of total runtime.

The aggregation of sequence strings into a contiguous buffer takes at least 374 ms, which is the major contributor to the overall runtime of GASAL, ADEPT, and WFA-GPU. In addition, WFA-GPU is spending a significant amount of time in other steps (not shown in the table) due to additional CPU work which looks like initialization of other data. In contrast, Accelign can accept a contiguous sequence buffer as input, avoiding additional memory copies.

The next step involves transferring the data from CPU RAM to GPU VRAM. With observed fast transfer rates of up to 350 GB/s due to Grace Hopper’s NVLink interconnect, the data transfer does not pose a bottleneck compared to the duration of compute kernels. However, on systems with a traditional PCIe interconnect like PCIe 5.0 ($\sim 50$ GB/s), performance can be limited by data transfer rate, rather than compute performance. For example, we have measured an alignment kernel duration of 35 ms when executing the short read benchmark on system S1 with RTX PRO 6000, but the data transfer with 50 GB/s would take around 60 ms.

The total pipeline performance using our library can further be improved by using CUDA streams to overlap CPU-GPU transfers with GPU kernels, yielding a total of 3.51 TCUPS including CPU and GPU work as well as data transfers. CUDA streams are also supported by GASAL2 and WFA-GPU to overlap different stages within the pipeline.

Besides real-world short-read Illumina data, we also evaluated performance on a real-world Nanopore dataset^3^ using the same benchmark setup. We replicated the data 16 times to obtain our benchmark set comprising 199,632 sequence pairs with a (minimum, maximum, mean, median) length of 40, 1122, 806, and 940, respectively, with approximately $146 \cdot 10^9$ DP cells to be computed. We repeated above benchmark using this dataset. Accelign used the short2 datatype instead of half2 to support a greater alignment score range at similar performance than half2. The best total achieved throughput of the tools is presented in Table 4.

**Table 4:** Best total TCUPS for the second pipeline stage for the Nanopore dataset

GPU libraries				CPU libraries						
Accelign	GASAL2	ADEPT	WFA	Parasail	Seqan	BSAlign	EdLib	KSW2	WFA2	A*PA2
3.78	1.19	–	0.34	0.02	0.03	0.11	1.23	0.09	0.62	0.72

On this dataset, Accelign achieves a kernel performance which is similar to the performance of short reads alignment. However, due to the quadratic complexity of sequence alignments, the fraction of alignment time compared to the total runtime increases, resulting in improved total throughput. A similar observation can be made for GASAL2 because overheads from buffer preparations become less significant. On the other hand, WFA-GPU shows performance degradations which are caused by an increased fraction of alignments being computed on the CPU, even after relaxing the corresponding maximum error threshold. ADEPT is unable to process the dataset due to alignment kernel launch errors caused by increased sequence lengths. For the CPU tools, note that the performance of both Edlib and A*PA2 increases compared to the short Illumina reads. This indicates the importance of banded alignments with increasing sequence lengths and explains why banded alignments may be the preferred choice over a full DP matrix calculation.

Together, the two benchmarks highlight that Accelign is suitable for computation of exact alignment scores of sequences of multiple sequencing technologies with different length distributions. However, computing the full DP matrix comes at high computational cost, and heuristic approaches like banded alignments, for example, are typically more efficient for long read data at the cost of potentially inexact alignment results.

### Protein database scanning

The second case study focuses on the topic of similarity search. Given a database of reference sequences and a query sequence, the goal is to find those reference sequences which are the most similar to the query sequence. One typical use-case is protein homology search as performed, for example, by BLASTP. Accelign supports database searches, for example via one-to-all alignment kernels utilizing a PSSM. A suitable alphabet size can be set to handle DNA/RNA, or proteins.

To benchmark our performance, we have set up the following experiment. The UniRef50 protein database (Release 2025_03 consisting of 20,080,454,799 amino acids in 70,198,728 sequences with largest (average) sequence length of 45,354 (282)) was used as reference. Similarity search was performed for a set of 20 real-world protein queries with sequence lengths varying between 144 and 5, 478. The employed alignment was a one-to-all PSSM local alignment with affine gap penalties (gap open $= -11$, gap extend $= -1$) and the BLOSUM62 substitution matrix (BLASTP default settings).

As a first step, we compute all alignments in reduced precision using fast packed 16-bit or 8-bit computations. This gives the exact alignment score for the majority of reference sequences. However, a small fraction of scores could be incorrect due to the limited resolution. Thus, we inspect the obtained alignment scores and recompute each score that is not flagged as exact using 32-bit calculations.

Since Accelign is designed as a kernel-level API, its users may directly use the functions on multiple GPUs. We also demonstrate this capability in the context of the current benchmark. One approach to a multi-GPU database scan is to partition the database across the different GPUs, search the input query in the database partitions of all GPUs simultaneously, and merge the per-GPU result lists into a final result. In our case, we decided to partition the database such that each GPU computes a similar number of DP matrix cells. More advanced partitioning schemes may be used in real-world applications to achieve better load balancing. For example, it may be beneficial to have a similar number of alignments per GPU in addition to a similar number of matrix cells. In any case, our trivial partitioning is already sufficient to demonstrate the library capability and benefits of using multiple GPUs.

We compare the performance of Accelign to the GPU-based CUDASW++4.0 and the CPU-based BLASTP (version 2.17.0+) using 128 CPU threads on S1. For all programs, the reference database was first converted into a binary format suitable for processing. For CUDASW++4.0 and Accelign benchmarks, the resulting binary database fits completely into GPU memory. The duration of this pre-processing step and database transfer from CPU memory to GPU memory is excluded from our evaluation.

Table 5 reports the performance of CUDASW++4.0 and Accelign in terms of TCUPS for each of the 20 queries.

For Accelign we are evaluating three configurations per query: high-level API (HL) and customized (C), both making use of half2 alignments in the first pass, as well as a configuration (U8) that performs uint8x4 alignments in the first pass. The customized setting uses a configuration tailored towards the specific query length; e.g., the tilesize $464=16 \cdot 29$ was introduced which avoids out-of-bounds computations for query 4. Using a single GPU on S1 Accelign achieves an average performance 6.44 TCUPS with high-level API mode, 6.73 TCUPS in customized mode, and 12.33 TCUPS using 8-bit integers, outperforming CUDASW++4.0 (6.38 TCUPS on average) and BLASTP with 128 CPU threads (2.10 TCUPS on average). Furthermore, using both GPUs on S1, our library with a custom config achieves a speedup of $88\%$–$96\%$ over the single-GPU case, demonstrating efficient scaling to a multi-GPU setting.

**Table 5:** Database scan performance (TCUPS) with real protein queries. HL: indicates higher-level library implementation. C: indicates implementation with custom library config. U8: indicates uint8x4 datatype. BLASTP achieves an average of 2.10 TCUPS over all queries. Speedups are given over CUDASW4

Query	Length	CUDASW4	Accelign				Speedup		
			HL	C	C (2 GPUs)	U8	HL	C	U8
0 (P02232)	144	5.62	6.27	6.27	11.81	11.60	1.12	1.11	2.06
1 (P05013)	189	5.90	7.04	7.04	13.27	13.46	1.19	1.19	2.28
2 (P14942)	222	6.00	7.17	7.16	13.96	13.66	1.20	1.19	2.28
3 (P07327)	375	6.25	6.93	6.93	13.59	13.65	1.11	1.11	2.19
4 (P01008)	464	6.32	6.59	7.07	13.85	13.22	1.04	1.12	2.09
5 (P03435)	567	6.37	6.22	7.09	13.86	11.93	0.98	1.11	1.87
6 (P42357)	657	6.41	5.92	6.73	12.64	11.77	0.92	1.05	1.84
7 (P21177)	729	6.43	6.50	6.50	12.36	12.92	1.01	1.01	2.01
8 (Q38941)	850	6.46	6.49	6.50	12.34	12.82	1.01	1.01	1.99
9 (P27895)	1,000	6.48	6.85	6.85	12.99	13.58	1.06	1.06	2.10
10 (P07756)	1,500	6.53	6.47	6.82	13.32	12.37	0.99	1.05	1.90
11 (P04775)	2,005	6.55	6.61	6.83	13.35	12.36	1.01	1.04	1.89
12 (P19096)	2,504	6.57	6.20	6.76	13.13	11.49	0.94	1.03	1.75
13 (P28167)	3,005	6.58	6.58	6.81	13.31	12.22	1.00	1.03	1.86
14 (P0C6B8)	3,564	6.59	6.55	6.84	13.24	12.26	0.99	1.04	1.86
15 (P20930)	4,061	6.57	6.57	6.80	13.15	12.14	1.00	1.03	1.85
16 (P08519)	4,548	6.56	5.86	6.66	12.72	11.04	0.89	1.01	1.68
17 (Q7TMA5)	4,743	6.53	6.05	6.24	11.90	11.57	0.93	0.96	1.77
18 (P33450)	5,147	6.47	6.17	6.14	11.70	11.88	0.95	0.95	1.84
19 (Q9UKN1)	5,478	6.38	5.84	6.49	12.38	10.66	0.92	1.02	1.67

### HIV read mapping

Aligners such as BWA and Bowtie2 [37] do not construct a complete DP matrix between the two sequences aligned, but instead rely on finding short exact matches or seeds first, followed by local alignment extension from these seeds. This is computationally efficient as long as exact seeds can be found between the two sequences, which can be challenging for evolutionary divergent sequences. The Human Immunodeficiency Virus (HIV) is one of these challenging examples, with diversity reaching up to 20% within the same subtype and over 30% between different subtypes [38]. This higher diversity leads to fewer matched seeds and alignments.

To showcase that an optimal aligner can perform better in such biological cases, we simulated 500,000 short, single-end reads from an HIV Subtype O assembly (accession number KU168292) retrieved from the Los Alamos National Laboratory HIV database. We used the ART Illumina simulator [39] v.2.5.8 with the MiSeq Version 3 setting (-ss MSv3 -l 250) to produce the simulated sequences. We aligned the simulated sequences against the HIV reference genome HXB2 (accession number K03455) using both BWA-MEM v.0.7.19 and Accelign. For both aligners, we used a match score of 1, a mismatch penalty of 3, a gap opening penalty of 6, a gap extension penalty of 1 and a minimum score cutoff of 30. Accelign was able to align 179,301 sequences compared to BWA that aligned only 77,992 sequences; all sequences aligned by BWA were also aligned by Accelign.

**Fig. 6:**
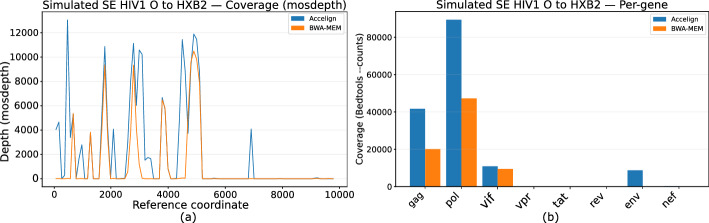
Results for the simulated single-end reads from the HIV1 O subtype aligned against the HXB2 reference using both Accelign and BWA. Panel (**a**) shows the average coverage over the reference genome for both Accelign (blue) and BWA (orange). Panel (**b**) shows the coverage counts for each gene in the HIV reference genome. Similar to (**a**), we see that Accelign was able to align many more sequences for certain genes, and align sequences against the envelope protein, where BWA was not able to

Figure 6(a) shows the coverage of both aligners over the HXB2 genome, with the depth calculated over 100-bp bins using mosdepth v.0.3.10. From the plot, we can see that Accelign was able to align many more sequences in the same regions and also align sequences to regions where BWA was not able to. Specifically, aligning sequences to the envelope (gp120) protein containing the highly variable V1-V5 loops, which are crucial in evading immunity [40]. Figure 6(b) plots the coverage counts for each gene in the reference; the counts were calculated using Bedtools version 2.31.1 with the --counts flag. This bar plot further highlights how Accelign was able to align many more sequences to genes, especially the envelope protein.

We used the following workflow to compute the best mapping with Accelign. First, we employed a 16-bit score-only local affine alignment using a substitution matrix to align both the forward read and its reverse complement against the reference. With the given sequence lengths and scoring scheme, the optimal local alignment score is guaranteed to be less than 2048 which removes the need of re-computing scores in greater precision. Furthermore, orienting reads along the horizontal axis of the DP matrix allows to process the resulting tall matrix using a single matrix tile without temporary storage.

Next, for each read where at least one of the two directions reaches the minimum score cutoff, we identify the direction with the best score. While we do not provide traceback functionality in our library, it can nonetheless be used to speed up subsequent traceback computations. Specifically, for the identified set of best alignments, we recomputed the alignments with our kernel that finds the start and end positions of the traceback in addition to the alignment score. The resulting positions as well as alignment scores are subsequently transferred to the CPU. As a final step, Block Aligner [41] is used to compute the tracebacks of the sub-sequences given by the start and end positions.

On system S1 with 128 threads, BWA reports a processing time of 0.78 seconds (M::mem_process_seqs), whereas our implementation takes 0.5 seconds for GPU alignments and 0.2 seconds for CPU-sided tracebacks with Block Aligner, including all data transfers between CPU and GPU. To summarize, both programs require a similar amount of time for the core alignment step but BWA produces less mapped reads.

## Conclusion and discussion

The continually increasing volume of sequence data in the life sciences results in a growing demand for high-throughput sequence processing pipelines. Pairwise sequence alignment is a major contributor to runtimes of many bioinformatics pipelines due to the associated quadratic time complexity. While faster heuristics exist for certain use cases, computing optimal alignments by means of DP is still important for a variety of applications.

We have presented Accelign, a GPU-accelerated open-source pairwise sequence alignment library. At its core it provides a low-level API to high-performance alignment kernel functions for different types of input data, scoring schemes and alignment types. The associated parallelization scheme is based on a common wavefront pattern using warp intrinsics for fast inter-thread communication that minimizes the overall executed instructions using in-register computation in order to unlock the processing power of modern GPU architectures. We provide accelerated kernels for local, global, and semi-global alignment, allowing for the computation of optimal alignment scores with various scoring schemes (linear or affine gap penalties, substitution matrix or PSSM) and alphabets (e.g. DNA/RNA, proteins) supporting one-to-one, one-to-all, and all-to-all use cases on a single or on multiple GPUs. In addition, start/end positions of optimal alignments can be provided. Computing detailed tracebacks is currently not supported, but based on start/end positions highly tuned CPU-based implementations for traceback of global alignments such as BlockAligner could efficiently be used for this task.

Our analysis demonstrates that the current CPU-based traceback implementation is suitable when processing only a small fraction of high-scoring alignments. However, this approach becomes a computational bottleneck in scenarios requiring extensive traceback computations. To address this limitation, we plan to propose GPU-accelerated traceback as a key extension to Accelign as part of our future work, necessitating careful architectural trade-offs. Storing the traceback matrix in on-chip shared memory (16 KB capacity) accommodates sequences $\le 128$ bp (1 byte/cell) but severely restricts concurrent alignment throughput per SM. While multi-warp alignment strategies could improve hardware utilization, they introduce inefficiencies through reduced per-thread computation and intra-warp synchronization overhead. On the other hand, using off-chip memory may inhibit performance due to memory bandwidth limitations. Note that even storing only the primary DP matrix in global memory would already slowdown our algorithm: with device memory bandwidth of 1 TB/s, storing one byte per cell would limit the performance to only 1 TCUPS. Promising are therefore approaches that trade-off global memory accesses with additional computation, such as adaptions of the Hirschberg algorithm (used in SW# [16] for GPU-enabled genome scale alignments) or gridded traceback (used in $\hbox {G}^3$SA [35]). Lastly, algorithmic design choices will depend on the ratio between compute throughput and memory bandwidth of the target GPU.

Our performance evaluation shows how Accelign can be used to accelerate a number of case studies: short read alignment, protein database scanning, and HIV read mapping. The associated performance evaluations show that it outperforms prior GPU-based libraries (GASAL2, ADEPT) and CPU-based libraries (SeqAn, BSalign, Parasail, EdLib, KSW2) as well as specialized GPU implementations (WFA-GPU, CUDASW++4.0). Our evaluation also demonstrates that speed can further increase by using newer GPU generations and by using multiple GPUs connected to the same host.

Another important use case could be the area of aligning long, noisy sequences of long reads generated by Oxford Nanopore or PacBio HiFi sequencers, which can produce significantly longer reads than Illumina. Therefore, several optimization efforts have addressed this use case by reducing the search space; e.g. through different types banding or X-drop heuristics. This can limit the amount of cells computed in the DP matrix drastically for long reads. Currently, our library is able to process reads of arbitrary lengths, but only supports the computation of optimal alignments by means of calculating the full DP matrix. Although this is done in an efficient manner, algorithms implementing above heuristics are likely to be faster in practice due to the vast amounts of skipped computations.

The final issue that often comes up when discussing GPU-based computing is cost efficiency. Because the total cost of high-performance processing power is strongly influenced by factors such as operational expenses and the specific infrastructure of an institution, it is rarely possible to assign a single “normalized” price. Nevertheless, we present a brief cost analysis of our benchmark programs using the AWS hourly rates that were current in May 2026. For the CPU baseline we used an c6a.16xlarge instance, which features a third-generation AMD EPYC processor (64 vCPUs, 128 GB RAM) which is similar to the hardware employed in our CPU benchmarks on System S1. For the GPU comparison we selected a g7e.2xlarge instance equipped with one RTX PRO 6000 Blackwell Server Edition, 8 vCPUs and 64 GB RAM. In the US-East (Ohio) region the hourly charges are 2.44 dollars for the CPU host and 3.36 dollars for the GPU host. In the short-read benchmark, WFA2 achieves 1.74 TCUPS on the CPU, which corresponds to 0.71 TCUPS per dollar. Accelign on the RTX PRO 6000, limited by data-transfer time, is estimated to reach roughly 3.3 TCUPS, or 0.98 TCUPS per dollar. For the protein database scan workload, Accelign (8-bit) delivers 12.33 TCUPS while BLASTP reaches 2.1 TCUPS, which translate to 3.66 TCUPS per dollar and 0.86 TCUPS per dollar, respectively. This shows that our benchmark programs are more cost efficient when executing with Accelign on the GPU instead of using a CPU-based library.

While the simple high-level library functions can be used to immediately accelerate applications without additional tuning efforts, the best performance might only be achieved by a custom configuration tuned towards a specific application. Our grid-search was only performed on a subset of possible matrix tile sizes. Another opportunity for improvement lies in the choice of matrix tile size for multi-tile processing depending on the sequence length. Ideally, we would need to consider the actual performance values of different tile sizes, which could be included in future releases. Based on our generic parallelization strategy the library can be easily extended for other DP-based algorithms such as dynamic time warping for Nanopore signals, the Viterbi algorithm for profile HMMs, or profile-to-profile alignments.

## Data Availability

Accelign is available at https://github.com/fkallen/Accelign. The datasets used for evaluation can be found at https://zenodo.org/records/19855732. These datasets were originally obtained from https://www.ebi.ac.uk/ena/browser/view/SRR1766553 (SRR1766553), https://ftp.uniprot.org/pub/databases/uniprot/previous_releases/release-2025_03/ (UniRef50 Release 2025_03), https://www.hiv.lanl.gov/components/sequence/HIV/asearch/query_one.comp?se_id=KU168292 (O.FR.2005.LA49RBF189.KU168292), and https://www.ncbi.nlm.nih.gov/nuccore/K03455 (K03455).

## Footnotes

1 https://docs.nvidia.com/cuda/cuda-c-best-practices-guide/index.html#instruction-optimization.

2 https://www.ebi.ac.uk/ena/browser/view/SRR1766553

3 https://github.com/Daniel-Liu-c0deb0t/block-aligner/releases/download/v0.0.0/real.ont.b10M.txt.gz
